# Polycystin-1 Mediates Mechanical Strain-Induced Osteoblastic Mechanoresponses via Potentiation of Intracellular Calcium and Akt/β-Catenin Pathway

**DOI:** 10.1371/journal.pone.0091730

**Published:** 2014-03-11

**Authors:** Hua Wang, Wen Sun, Junqing Ma, Yongchu Pan, Lin Wang, Weibing Zhang

**Affiliations:** 1 Institute of Stomatology, Nanjing Medical University, Nanjing, China; 2 The Research Center for Bone and Stem Cells, Department of Anatomy, Histology and Embryology, Nanjing Medical University, Nanjing, China; Ohio State University, United States of America

## Abstract

Mechanical regulation of bone formation involves a complex biophysical process, yet the underlying mechanisms remain poorly understood. Polycystin-1 (PC1) is postulated to function as a mechanosensory molecule mediating mechanical signal transduction in renal epithelial cells. To investigate the involvement of PC1 in mechanical strain-induced signaling cascades controlling osteogenesis, PKD1 gene was stably silenced in osteoblastic cell line MC3T3-E1 by using lentivirus-mediated shRNA technology. Here, our findings showed that mechanical tensile strain sufficiently enhanced osteogenic gene expressions and osteoblastic proliferation. However, PC1 deficiency resulted in the loss of the ability to sense external mechanical stimuli thereby promoting osteoblastic osteogenesis and proliferation. The signal pathways implicated in this process were intracellular calcium and Akt/β-catenin pathway. The basal levels of intracellular calcium, phospho-Akt, phospho-GSK-3β and nuclear accumulation of active β-catenin were significantly attenuated in PC1 deficient osteoblasts. In addition, PC1 deficiency impaired mechanical strain-induced potentiation of intracellular calcium, and activation of Akt-dependent and Wnt/β-catenin pathways, which was able to be partially reversed by calcium ionophore A23187 treatment. Furthermore, applications of LiCl or A23187 in PC1 deficient osteoblasts could promote osteoblastic differentiation and proliferation under mechanical strain conditions. Therefore, our results demonstrated that osteoblasts require mechanosensory molecule PC1 to adapt to external mechanical tensile strain thereby inducing osteoblastic mechanoresponse, partially through the potentiation of intracellular calcium and downstream Akt/β-catenin signaling pathway.

## Introduction

Mechanical loading is an important epigenetic factor for the regulation of skeletal tissue regeneration [1]. Increased mechanical loading stimulates osteoblastic differentiation and proliferation thereby resulting in bone formation [2]. In contrast, low level or absence of mechanical loading leads to either no response or reduced bone synthesis [3], [4]. Osteopenia or osteoporosis is in part caused by lack of physiological mechanical loading [5]. Therefore, understanding the physiological mechanisms of bone how to adapt to mechanical stimuli should contribute greatly to prevent bone loss [6].

Mechanical regulation of bone formation involves a complex biophysical process, including the perception of extracellular mechanical stimuli applied, their conversion into intracellular biochemical cascades and ultimately adaptive responses of bone cells [7], [8]. Mechanosensors sensing extracellular mechanical stimuli is a critical step of this process. An increasing number of mechanosensors have been identified, such as cell-matrix adhesion proteins, cell cytoskeleton, and primary cilia [9]. However, the molecular mechanism how original mechanosensory molecule perceives mechanical signals thereby transforming into biochemical signals still remains poorly understood.

Polycystin-1(PC1), encoded by polycystic kidney disease gene 1 (PKD1), has been identified in patients with autosomal dominant polycystic kidney disease (ADPKD). PC1 and polycystin-2 (PC2) form a Ca^2+^-permeable mechanosensitive ion channel, and mediate mechanosensory signal transduction in renal epithelial cells [10], [11], [12]. Lack of functional PC1 displays much less Ca^2+^ influx in response to mechanical stimuli [13]. Moreover, PC1-deficient mice exhibit multiple developmental defects, including skeletal and vascular abnormalities [14], [15], [16]. Recent researches show that PC1 plays an important role in bone development through Runx2-dependent signaling cascade [17], [18]. Conditional deletion of PC1 results in impaired mechanical load-induced bony anabolic response in vivo [19]. Therefore, PC1 may play a key role in the mechanotransduction process regulating bone growth under mechanical loading conditions.

The Wnt/β-catenin pathway plays a key role in bone-cell differentiation and proliferation [20], [21], [22], [23]. Glycogen synthase kinase-3β (GSK-3β) is originally identified as a serine/threonine kinase, which induces the degradation of β-catenin. Intracellular Ca^2+^ is linked to the regulation of Akt/protein kinase B activity [24], [25] which is known to directly phosphorylate and thereby inactivate GSK-3β [26]. In addition, the earliest events in osteoblastic mechanotransduction are a rapid influx of extracellular Ca^2+^ and mobilization of intracellular Ca^2+^ [25], [27]. PC1 has been reported to be associated with the regulation of intracellular Ca^2+^ in response to mechanical stimuli [28]. Therefore, we propose a mechanism that PC1 as an initial mechanosensory molecule perceives mechanical strain and then mediates mechanical strain-induced osteoblastic mechanoresponses through intracellular signaling cascades involving interactions between intracellular Ca^2+^, Akt, GSK-3β and β-catenin.

In this study, we used lentivirus-mediated shRNA technology to stably silence PKD1 gene in MC3T3-E1 cells. Then we examined the effects of PC1 on mechanical strain-induced osteoblastic mechanoresponse and related signaling cascades. Collectively, we demonstrated that PC1 is required for the mechanical strain-induced osteoblastic mechanoresponse, associated with intracellular calcium and Akt/GSK-3β/β-catenin signaling pathway.

## Materials and Methods

### Reagents

Fetal bovine serum (FBS), α-minimum essential medium (α-MEM), penicillin/streptomycin, L-glutamine and trypsin/EDTA were purchased from Gibco. GSK-3β inhibitor Lithium Chloride (LiCl), Akt1/2 kinase inhibitor Akti-1/2, and calcium ionophore A23187 were purchased from Sigma-Aldrich. Antibody against PC1 (sc-25570) was purchased from Santa Cruz Biotechnology. Antibodies against active β-catenin (05-665), Osteocalin (OCN) (AB10911) and β-actin (04-1116) were purchased from Merck Millipore. Antibodies against anti-Ser-473 phospho-Akt (4060), total Akt (4685), anti-Ser-9 phospho-GSK-3β (9366) and anti-GSK-3β (9832) were purchased from Cell Signaling Technologies. Antibodies against Runx2 (ab76956), Osterix (Osx) (ab22552), and Osteopontin (OPN) (ab8488) were purchased from Abcam. The antibody against total β-catenin (610154) was purchased from BD Biosciences. For western blotting, the primary antibodies were detected using horseradish peroxidase-linked anti-mouse (04-18-06) or anti–rabbit (04-15-06) conjugates as appropriate (Kirkegaard & Perry Laboratories, Inc.). For immunocytochemistry, secondary antibody Fluorescein (FITC)-conjugated AffiniPure Goat Anti-mouse IgG (H+L) (BS50950) (Bioworld Technology, Inc.) and Alexa Fluor 594 Goat Anti-Mouse IgG (H+L) (A11005) (Invitrogen Co.) were used as appropriate.

### Cell culture

The mouse osteoblastic cell line MC3T3-E1 was purchased from the Cell Bank of the Chinese Academy of Sciences (Shanghai, China). Cells were cultured in α-MEM containing 10% FBS, 1% glutamax and 1% penicillin/streptomycin, and maintained at 37°C and 5% CO_2_. Cells used for quantification of alkaline phosphatase activity were grown in differentiation media (a-MEM with 10% FBS, 0.1 μM dexamethasone, 10 mM β-glycerophosphate and 50 μM ascorbic acid). The medium was refreshed every other day. The following pharmacological agents and their concentrations were used on cells: LiCl; 20 mM, Akti-1/2; 40 μM, and A23187; 200 μM.

### Lentiviral shRNA Vector Construction and Transfection

Four target shRNAs against mouse PKD1 gene were designed as follows:

shRNA#1:5′-GCATCTCACTGTCCCTCAACT-3′,

shRNA#2:5′-GCAGACTTCCATATTACTTCC-3′,

shRNA#3:5′-GCGGATGAACAAGATGCATGG-3′, and shRNA#4:5′-GCATATTCCCACTGGCATTGG-3′. Oligonucleotides encoding shRNA sequences and one negative control sequence (5′-TTCTCCGAACGTGTCACGT-3′, which showed no significant homology to any mouse gene) were synthesized and annealed into double strands [29]. Double-stranded DNAs were inserted into slow virus interference vector (pGLVH1/GFP+Puro, encoding green fluorescent protein (GFP), the lentiviral frame plasmid was supplied by Genechem Co. Shanghai, China) to generate PKD1 shRNA#1, shRNA#2, shRNA#3, shRNA#4, or control shRNA expression vector. The accuracy of the inserted into the recombinants was verified by restriction enzyme analysis and sequencing. Then ViraPower™ Lentiviral Expression System (Invitrogen) was used to generate lentivirus supernatants from 293T cells. In brief, recombinant non-integrative lentiviral vectors were produced by co-transfecting 293T cells with the lentivirus expression plasmid and packaging plasmid (pHelper 1.0 including gag/pol and pHelper 2.0 including VSVG) using Lipofectamine 2000 (Invitrogen). Twenty-four hours after initiating transfection, the plasmid–lipofectamine solution was removed, and the cell growth medium without antibiotics was added. The lentivirus-containing supernatants were harvested and concentrated 48 h and 72 h post-transfection. After transfection, the viral titer was determined by counting GFP-positive cells and diluted to 10^8^ TU/ml ultimately. MC3T3-E1 cells were transfected with appropriate dilutions of lentivirus supernatants, and the transduction efficiency was observed under a fluorescent microscope. Forty-eight hours after transfection, the cells were cultured in cell growth medium containing puromycin (2.5 μg/ml) for 72 h, and subsequently (1.75 μg/ml) for one week, in order to establish stably transfected MC3T3-E1 cells. Interference efficiency was detected using quantitative real-time PCR and western blotting.

### Mechanical Strain and Preparation of Cell Extracts

For strain experiments, cells were plated on 6-well Bioflex Collagen I coated plates (Flexcell International, Hillsborough, NC) at a density of 5×10^4^ cells per well for proliferation studies and 1×10^5^ cells per well for signal transduction studies [30]. Cells were transferred to medium containing 0.1% FBS for the last 24 h before beginning experiments, and pre-incubated with pharmacological treatment for 1 h. Cyclic sinusoidal continuous tensile strain was applied (2% magnitude, 0.5 Hz) using the Flexcell FX-5000™ Tension System. Static control cells were cultured under identical conditions except mechanical strain. Immediately after the stretching was completed, whole cell extracts or nuclear lysates were prepared. Whole cell extracts were prepared according to the manufacturer's instructions (Keygen China), and nuclear lysates were collected using NE-PER extraction reagent (Pierce, Rockford, IL, USA) according to the manufacturer's protocol. Protein concentrations were determined using a Bio-Rad protein assay kit (Pierce, Rockford, IL, USA).

### RNA Isolation and Quantitative Real-time PCR

Cells were harvested in 1 ml TRIzol reagent (Invitrogen). Total RNA was isolated according to the manufacturer's protocol (Invitrogen) and treated with RNase-free DNase. The concentration and purity of the RNA samples were determined by the absorbance of RNA at 230, 260, and 280 nm, respectively. Reverse transcription reaction was performed on 1 μg of RNA using the First Strand Synthesis Kit (Takara, Bio, Otsu, Japan). Relative transcript levels were measured by quantitative PCR in 25 μl reaction volume using ABI Prism 7300 sequence detection system (Applied Biosystems, Foster City, CA, USA), according to the recommended protocol for SYBR-Green (Roche), and normalized with GAPDH levels. Sense and anti-sense primers (Invitrogen) used were listed in Table 1. Real-time RT-PCR reaction conditions were: 95°C for 30 s, followed by 40 cycles of 95°C for 5 s and 61°C for 31 s. The results were obtained using the comparative CT method and the arithmetic formula 2^−ΔΔCT^. Experiments were performed three times.

**Table 1:** Sense and anti-sense primers for Real-time PCR.

Genes	Accession no.	Forward(5′–3′)	Reverse(5′–3′)
M-PKD1	NM_013630	CGGACCCACTATTTCCTACACC	GGGCAGAACCCACCTCATT
β-catenin	NM_001165902	ATGGAGCCGGACAGAAAAGC	CTTGCCACTCAGGGAAGGA
Axin2	NM_015732	TGACTCTCCTTCCAGATCCCA	TGCCCACACTAGGCTGACA
Runx2	NM_009820	TTGACCTTTGTCCCAATGC	AGGTTGGAGGCACACATAGG
Osterix	NM_130458	ATGGCGTCCTCTCTGCTTG	TGAAAGGTCAGCGTATGGCTT
Osteocalcin	NM_007541	CTCACAGATGCCAAGCCCA	CCAAGGTAGCGCCGGAGTCT
Osteopontin	NM_001204203	AGCAAGAAACTCTTCCAAGCAA	GTGAGATTCGTCAGATTCATCCG
GAPDH	NM_008084	ACAGCCGCATCTTCTTGTGC	CACTTTGCCACTGCAAATGG

### Western Blot Analysis

Extracted proteins were separated by SDS–PAGE and immunoblotting was carried out as described before [2]. Briefly, primary antibodies were incubated overnight at 4°C. Then the membranes were rinsed with TBST (0.1% Tween-20 in 0.01 M TBS). The blots were incubated with appropriate secondary antibody at room temperature for additional 1 h. Protein bands were visualized using an enhanced chemiluminescence system (Supersignal West Pico Trial Kit, Pierce, Rockford), and the density of each band was quantified with a Fluor-S MultiImager (Bio-Rad). The experiment was repeated in triplicate.

### Immunofluorescence

After washing with PBS, cells were fixed in 4% paraformaldehyde and permeabilized in 0.25% Triton X-100/PBS at room temperature for 30 min. After washing with PBS, cells were blocked in blocking solution containing 2% normal goat serum for 1 h. Primary antibodies were incubated overnight at 4°C with diluted 1∶50 in blocking solution. After washing three times with PBS, appropriate fluorescent-labeled secondary antibody conjugates with appropriate dilution in PBS were performed for 1 h in darkness. Finally, nuclei were counterstained with DAPI (4′,6-diamidino-2-phenylindole, Invitrogen) for 5 min. Fluorescence microscopy was viewed using a Zeiss LSM 510 confocal microscope. Experiments were performed three times.

### Cytochemical staining for alkaline phosphatase (ALP)

Cells were incubated for 15 min at room temperature in 100 mM Tris maleate buffer containing 0.2 mg/ml naphthol AS-MX phosphate (Sigma) dissolved in ethylene glycol monomethyl ether (Sigma) as a substrate and fast red TR (0.4 mg/ml, Sigma) as a stain for the reaction product. After washing with distilled water and air drying, ALP-positive colony areas were measured by image analysis. Experiments were performed three times.

### Alkaline phosphatase activity assay

The ALP assay was carried out with a ALP kit (Sigma) as described previously [31]. Briefly, cells were lysed by lysis buffer and the supernatant was collected for ALP detection. First, in each well of a 96-well plate, 50 μl of alkaline buffer solution and 50 μl of stock substrate solution were added and mixed well. Secondly, 10 μl of sample was added into each well, mixed and incubated at 37°C for 15 min. Then, 110 μl of 0.5 M NaOH was added to stop the reaction and the A (absorbance) at 405 nm was obtained. Finally, ALP activity was calculated according to the standard curve (ALP activity = 18.904×A−0.282, Sigma units) and normalized on the basis of equivalent protein concentrations. The experiment was repeated in triplicate.

### Assay of Intracellular Calcium

The intracellular calcium was measured with fura-2-acetoxymethyl ester (Molecular Probes, Inc., Eugene, OR) according to the methods previously described [32]. Fura-2 AM is the cell-permeable acetoxymethyl (AM) ester form of Fura-2. Fura-2 exhibits a shift in absorption when bound to Ca^2+^ such that the emission intensity when illuminated with ultraviolet light increases with calcium concentration at a wavelength of 340 nm and decreases with calcium concentration at 380 nm in turn. The ratio of light intensity between the two wavelengths corresponds to calcium concentration.

The cells plated on 6-well Bioflex plates were incubated with 5 μM Fura-2/AM in Hepes-buffered saline for 30 min at 37°C, then washed with fresh α-MEM and 2% FBS prior to strain experiments. An inverted fluorescence microscopy was used to record calcium concentrations of con-shRNA and PKD1-shRNA cells one every 10 s under strain. Fluorescence intensities were recorded on the setting of excitation wavelengths 340 nm and 380 nm, emission wavelength 510 nm. The intracellular calcium concentrations was calculated from the Fura-2 fluorescence ratio (F340/F380) using linear regression between adjacent points on a calibration curve generated by measuring F340/F380 in six calibration solutions. Experiments were performed three times.

### Flow cytometry

Flow cytometry measurements were performed according to previous studies [33]. In brief, cells were harvested by exposure to trypsin/EDTA and centrifuged at 600 g for 3 min. Cell precipitates were washed twice with PBS and resuspended in 1 ml of physiological saline, fixed in 2 ml of cold dehydrated alcohol and stored at 4°C overnight. Then, each sample was washed again with PBS, and incubated with propidium iodide (100 mg/ml; Sigma) on ice for at least 30 min. Cell cycle fractions (G0/G1, S, and G2/M phases) were determined by FCM. The experiment was repeated three times.

### EdU Incorporation

Cells proliferation rate was detected using EdU (5-Ethynyl-2′-deoxyuridine) incorporation assays according to the manufacturer's protocol (Click-iT EdU proliferation kit, Invitrogen). Briefly, cells were subjected to mechanical strain for 2 h followed by incorporation with EdU (20 μM) for 1 h, and then processed for immunofluorescence as above. To assess proliferation by EdU staining, the percentage of cells stained positive was analyzed under 20× magnifications in four randomly chosen fields per plate. In each case, representative proliferation results are shown as the proportion of all cells in each field stained positive for EdU. Assays were performed three times using triplicate plates.

### Statistics

All experiments were repeated at least three times. Differences between treatment and control groups were analyzed using Student's t-tests. Values of P<0.05 were considered statistically significant. Results were expressed as fold change (mean ± SEM) compared to values for the control group.

## Results

### Assessment of Interference Efficiency

**Figure 1:**
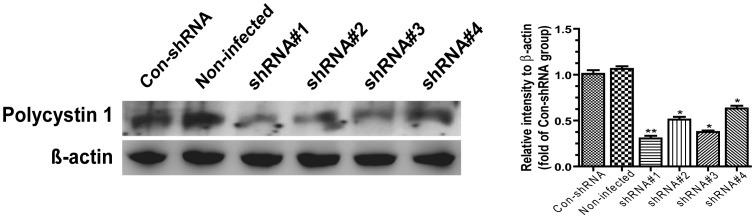
Efficiency of lentivirus-shrna interference as detected by western blot. The expression of PC1 in osteoblasts 72-infected and con-shRNA group. Compared with con-shRNA group, the levels of PC1 in lentivirus-shRNA#1, 2, 3 and 4 subgroups were reduced by 31.9%, 51.1%, 39.6%, and 63.2%, respectively. Each experiment was performed three times individually. Results are presented as the mean ± SEM, * indicates significantly different than controls (*, p<0.05; **, p<0.01; ***, p<0.001).

Compared with control shRNA cells, the PKD1 mRNA level in PKD1 shRNA#1 cells declined 71% by real-time quantitative PCR analysis, and the PC1 protein level reduced 69% by western blot analysis. Compared with other target shRNAs, the interference efficiency of shRNA#1 was the highest. Thus, we chose transfected PKD1 shRNA#1 cells for the further cellular function and signaling pathway experiments. Meanwhile there was no difference between non-infected cells and transfected control shRNA cells, demonstrating that the transfection process did not affect cell behavior (Fig. 1, Table 2).

**Table 2:** Relative expression levels of PKD1 in different groups as detected by Real-time PCR.

	ΔCt(PKD1-GADPH')	Relative expression
Con-shRNA	3.88±0.09	1.000
Non-infected	3.80±0.08	1.064
lentivirus-shRNA#1	5.60±0.14*	0.309
lentivirus-shRNA#2	4.42±0.18*	0.691
lentivirus-shRNA#3	5.15±0.09*	0.426
lentivirus-shRNA#4	4.96±0.17*	0.479

There were no significant differences between the non-infected and con-shRNA group with respect to the level of PKD1 mRNA. Compared with Con-shRNA group, the mRNA levels of PKD1 in lentivirus-shRNA#1, 2, 3 and 4 subgroups were reduced by 30.9%, 69.1%, 42.6%, and 47.9%, respectively. Each experiment was performed three times individually. Results are presented as the mean ± SEM, * indicates significantly different than controls (*, p<0.05; **, p<0.01; ***, p<0.001).

### Effects of Mechanical Strain on Osteoblast Differentiation and PKD1 Expression

**Figure 2:**
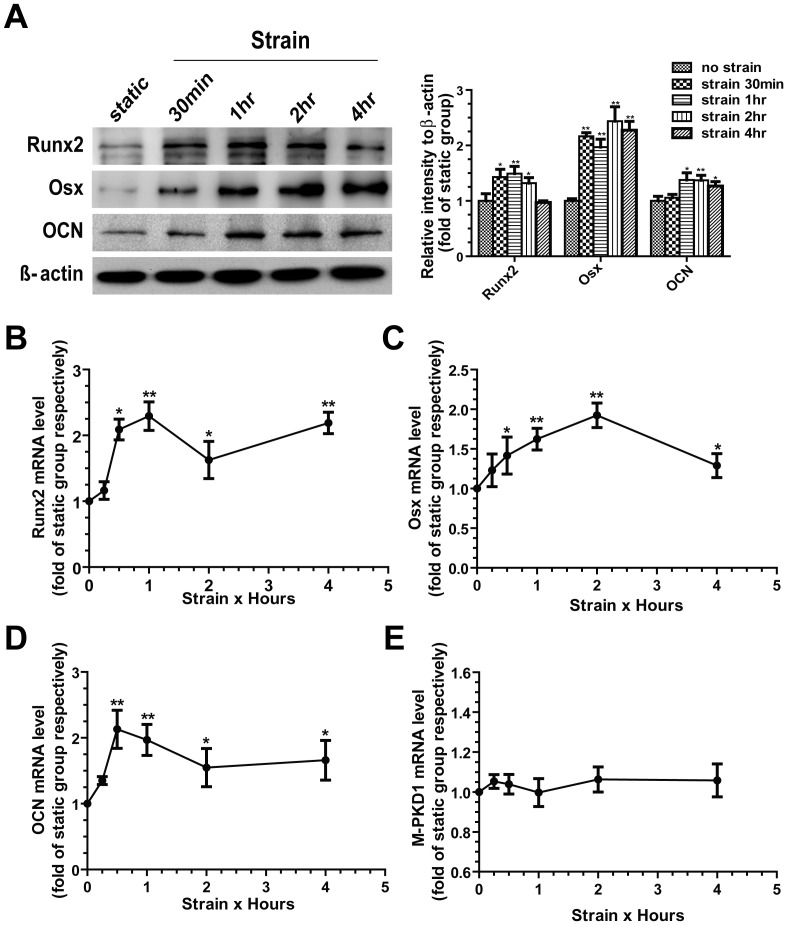
Effects of mechanical strain on osteoblast differentiation and PKD1 expression. (A) MC3T3-E1 cells were subjected to tensile strain for different time points (0–4 h), and the cellular protein were immunoblotted for Runx2, Osx, and OCN. Beta-actin was used as loading control. Total RNA was subjected to real-time quantitative PCR analysis for the gene expression of (B) Runx2, (C) Osx, (D) OCN and (E) PKD1. Messenger RNA expression is calculated as a ratio to the GAPDH mRNA level and expressed relative to non-strained control cells. Each experiment was performed three times individually. Results are presented as the mean ± SEM, * indicates significantly different than controls (*, p<0.05; **, p<0.01; ***, p<0.001).

Cells were subjected to a uniform continuous tensile strain according to previous studies [34], [35]. The certain osteogenic-related markers including Runx2, Osx, and OCN were investigated at both protein and mRNA level for various time points. Western blots showed a marked increase in the amount of Runx2 protein after strain, peaking at 1 h, as well as the up-regulation of OCN. The expression of Osx peaked at 2 h (Fig. 2A). Furthermore, total RNA was isolated and subjected to real-time quantitative PCR analysis. Runx2 mRNA expression peaked at 1 h after strain. Although Runx2 expression slightly decreased at 2 h, the level increased significantly at 4 h again (Fig. 2B). Consistent with Runx2, the mRNA expression of Osx and OCN peaked at 2 h and 1 h respectively (Fig. 2C, D).

Previous studies have shown that PC1 plays a key role in the mechanotransduction process in kidney epithelium cells [11], [13]. We investigated whether PC1 expression may be influenced by mechanical strain in osteoblasts. Unexpectedly, there was no significantly difference between non-strained cells and cells applied to continuous mechanical strain for each time point (Fig. 2E).

### Mechanical Strain-induced Osteoblast Differentiation Requires PC1

**Figure 3:**
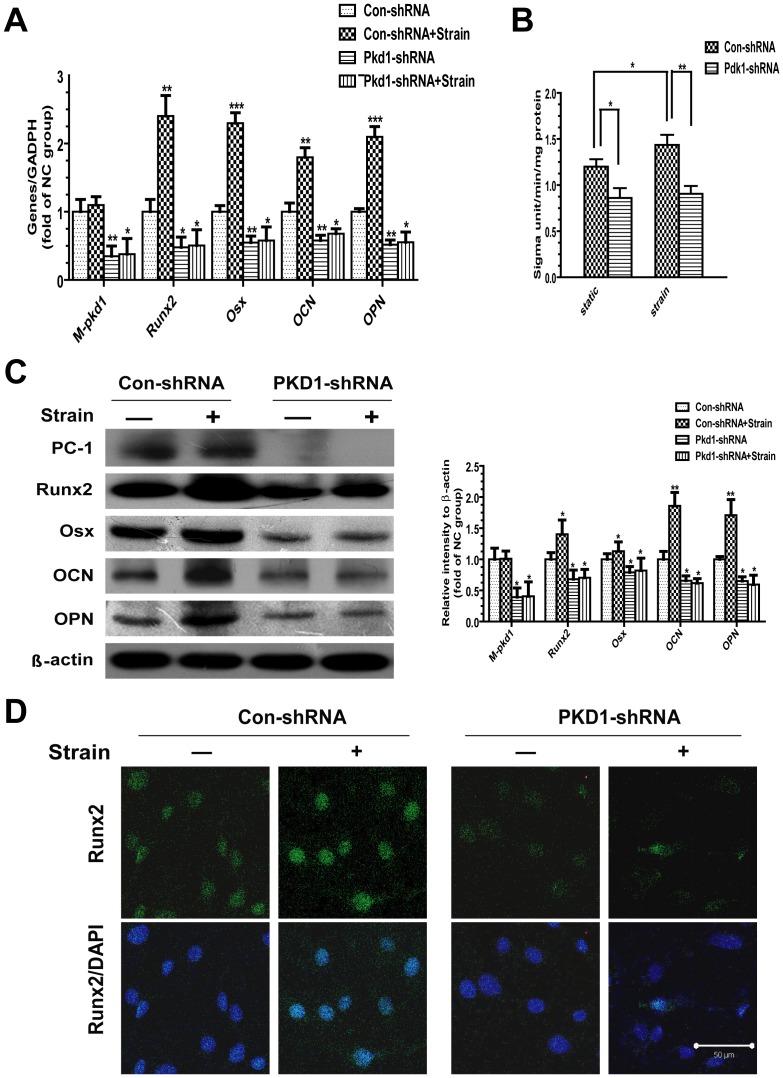
Strain-induced osteoblast differentiation needs PC1. (A) Effect of 1 h tensile strain on mRNA expression of PKD1, Runx2, Osx, OCN and OPN in con-shRNA and PKD1-shRNA cells. Messenger RNA expression was calculated as a ratio to the GAPDH mRNA level. (B) ALP concentrations in con-shRNA and PKD1-shRNA cells were examined after 1 h tensile strain (C) The protein levels of PC1, Runx2, Osx, OCN and OPN in con-shRNA and PKD1-shRNA cells after subjected to 1 h tensile strain. Beta-actin was used as loading control. (D) Runx2 levels were examined by immunofluorescence and confocal microscopy after 1 h tensile strain. Merged images of Runx2 (green staining) and nuclei counterstained with 4′,6-diamidino-2-phenylindole (DAPI, blue staining) were shown in the bottom. Scale bar, 50 μm. The experiment was repeated in triplicate. Results are presented as the mean ± SEM, * indicates significantly different than controls (*, p<0.05; **, p<0.01; ***, p<0.001).

Compared with control shRNA cells, stable silence of PKD1 gene caused a significant reduction in basal levels of PKD1 and osteoblastic differentiation marker genes, including Runx2, Osx, OCN and OPN. After 1 h strain, the osteoblastic differentiation maker genes were strongly up-regulated in control shRNA cells, but there were much less changes in PKD1 shRNA cells (Fig. 3A), as well as the ALP concentrations levels (Fig. 3B). To verify above results, western blots and immunofluorescence using an antibody against Runx2 were performed. The density measurement data of western blots revealed that the basal protein levels of PC1, Runx2, Osx, OCN and OPN reduced in PKD1 shRNA cells. The expressions of Runx2, Osx, OCN and OPN were significantly increased in strain group, whereas these up-regulations were no longer responsive to mechanical strain in PKD1 shRNA cells (Fig. 3C). In addition, immunofluorescence staining results also revealed that deletion of PC1 disrupted strain-induced up-regulation of Runx2 in PKD1 shRNA cells (Fig. 3D). These data suggest that PC1 may act as a chief mechanosensory molecule mediating strain-related osteoblastic differentiation.

### Changes of Proliferation in Response to Mechanical Strain in PC1 Deficient Osteoblasts

**Figure 4:**
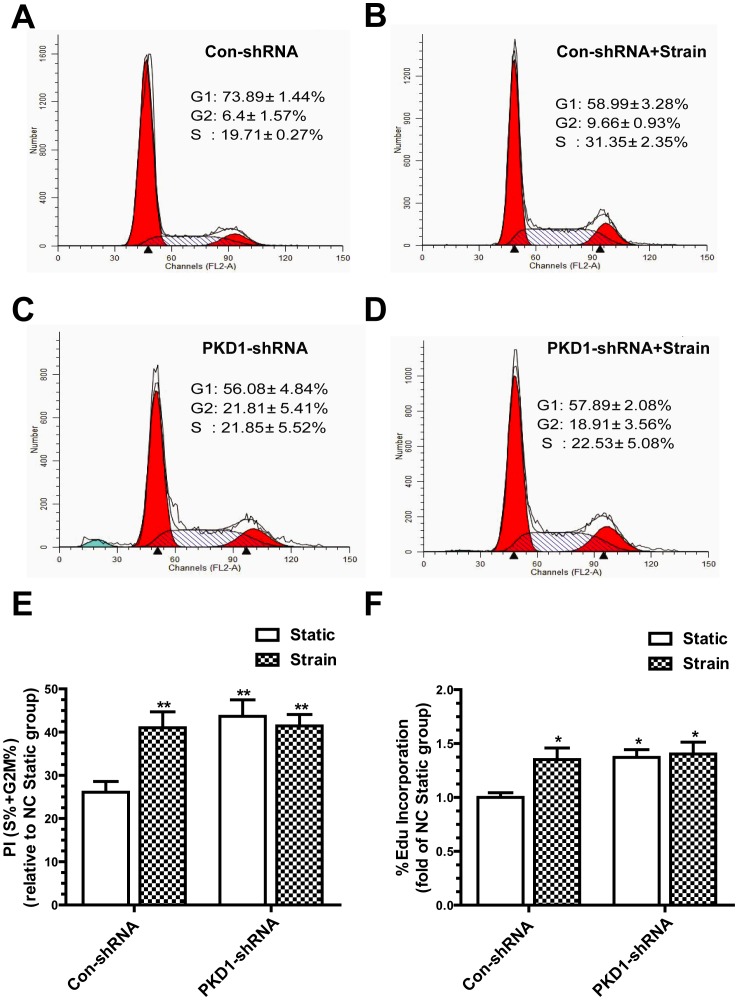
Changes of proliferation in response to mechanical strain in PC1 deficient osteoblasts. (A, B, C, D, E) Flow cytometry was assayed for proliferation index of con-shRNA and PKD1-shRNA cells after 2 h tensile strain. (F) EdU incorporation was assayed for proliferation rate of cells. Each experiment was performed three times individually. Results are presented as the mean ± SEM, * indicates significantly different than controls (*, p<0.05; **, p<0.01; ***, p<0.001).

After exposed to strain for 2 h, control shRNA cells showed a remarkable increase in the proliferation index compared with static control groups. However, there was no significant difference between the strained and static groups in PKD1 shRNA cells. Silence of PKD1 gene in osteoblasts resulted in the loss of the ability to sense external mechanical stimuli thereby promoting proliferation. Interestingly, deletion of PC1 leaded to a significant increase in basal proliferation index (Fig. 4A, B, C, D, E). To confirm above data, EdU incorporation assays were carried out [36]. Cells were subjected to 2 h mechanical strain followed by EdU incorporation for 1 h, whose data further demonstrated a similar result with flow cytometry assays (Fig. 4F).

### Mechanical Strain Induces Nuclear Accumulation of Active β-catenin

**Figure 5:**
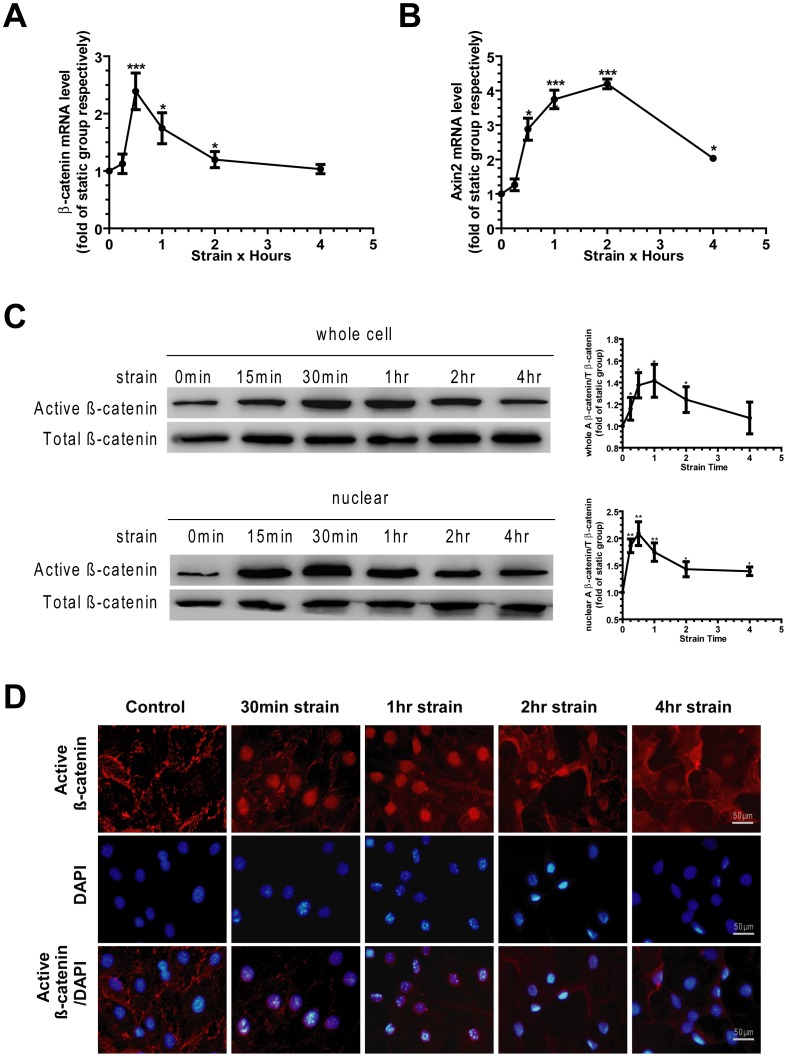
Mechanical strain induces nuclear accumulation of active β-catenin. (A) Levels of β-catenin gene were measured by real-time quantitative PCR. (B) Levels of Axin2 gene were measured by real-time quantitative PCR. (C) Western blots results showed levels of active β-catenin from whole cell lysates and nuclear fractionates. Total β-catenin (both phosphorylated and non-phosphorylated forms) was shown as a comparison. (D) Cellular distribution of active β-catenin (red staining) and nuclei counterstained with DAPI (blue staining) were determined by immunofluorescence and confocal microscopy. Scale bar, 50 μm. Each experiment was performed three times individually. Results are presented as the mean ± SEM, * indicates significantly different than controls (*, p<0.05; **, p<0.01; ***, p<0.001).

The expression of β-catenin gene was strongly up-regulated in strain groups, with a peak increase of over 2-fold at 30 min, and then gradually returned to base line by 4 h (Fig. 5A). The downstream targeting gene of β-catenin should be Axin2. Therefore, the expression of Axin2 gene was measured to detect β-catenin activity. Consistent with the rapid activity of β-catenin, a transient increase of Axin2 was detected after mechanical strain initiation, with a maximal increase of over 3-fold at 2 h compared with the control level (Fig. 5B).

Active (non-phospho) β-catenin is the stabilized form of β-catenin, which accumulates and then translocates into nuclei to activate downstream responsive target genes [37], [38]. Compared with static control level, active β-catenin activation occurred after mechanical strain. The active β-catenin of whole cell lysates peaked at 1 h, and gradually returned to base line by 4 h. Meanwhile, nuclear active β-catenin significantly increased after mechanical strain, with a maximal increased of 2-fold at 1 h compared with the static control level (Fig. 5C). Total β-catenin (both phosphorylated and non-phosphorylated forms) was shown as a comparison.

Immunofluorescence staining results revealed that there was only weak nuclear fluorescence for active β-catenin in static control cells. However after strain initiation, the fluorescence intensity of active β-catenin in nuclei was strongly increased. The nuclear active β-catenin accumulation remained high by 30 min to 1 h. By 4 h the pattern and intensity of fluorescence was similar to that in static control cells (Fig. 5D). These data suggest that mechanical strain induces nuclear translocation of active β-catenin, which is paralleled with strain-induced osteogenesis.

### Strain-induced Nuclear Translocation of Active β-catenin needs GSK-3β Inhibition and Akt-dependent pathway

**Figure 6:**
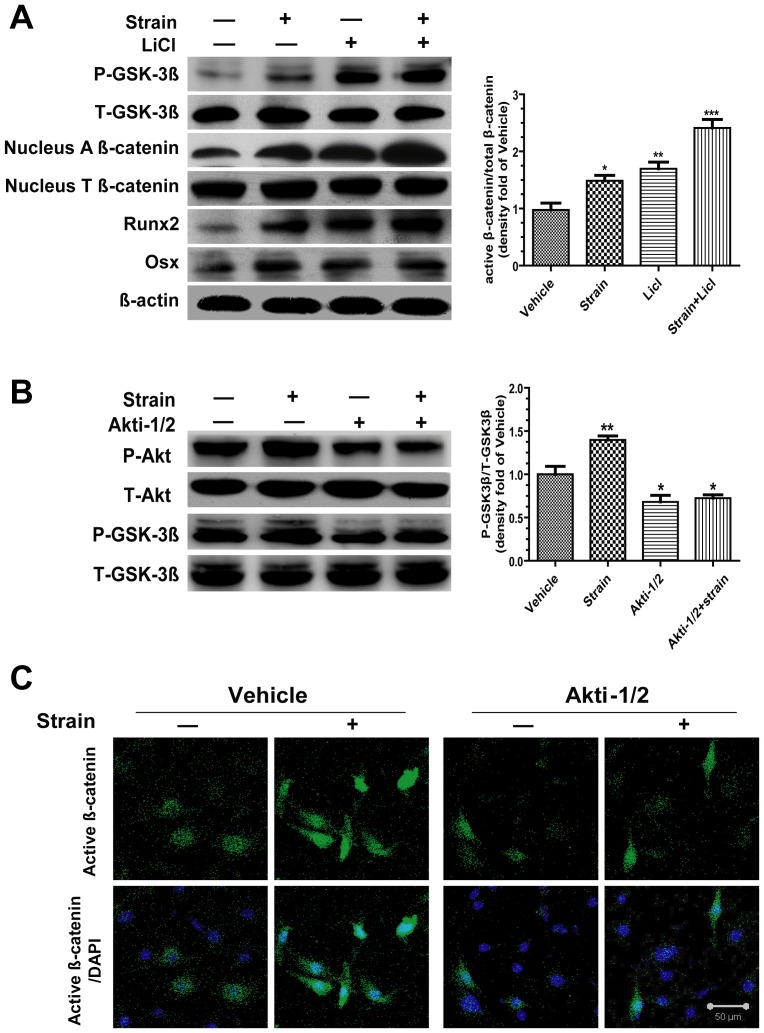
Strain-induced nuclear translocation of active β-catenin requires GSK-3β inhibition and Akt-dependent pathway. (A) Cells were pre-incubated with LiCl (20 mM) for 1 h and then subjected to1 h tensile strain. Western blots results showed levels of total and phospho-GSK-3β, nuclear total and active β-catenin, Runx2 and Osx. Runx2 and Osx were normalized to β-actin. Phospho-GSK-3β and active β-catenin were normalized to their respective total protein. The graph represented relative intensities of the active β-catenin bands normalized to total β-catenin, as measured by densitometry. (B) Cells were pre-incubated with Akti-1/2 (40 μM) for 1 h and then subjected to1 h tensile strain. Western blots results showed levels of the phosphorylation of Akt and GSK-3β. The graph represented relative intensities of phospho-GSK-3β bands normalized to total GSK-3β, as measured by densitometry. (C) Cells were pre-incubated with Akti-1/2 for 1 h and then subjected to tensile strain for 1 h before being fixed. Cellular distribution of active β-catenin (green staining) and nuclei counterstained with DAPI (blue staining) were determined by immunofluorescence and confocal microscopy. Scale bar, 50 μm. Each experiment was performed three times individually. Results are presented as the mean ± SEM, * indicates significantly different than controls (*, p<0.05; **, p<0.01; ***, p<0.001).

Western blots results showed that both LiCl and mechanical strain increased the phosphorylation of GSK-3β, as well as the expressions of Runx2 and Osx. Furthermore, LiCl enhanced the mechanical strain-induced nuclear accumulation of active β-catenin (Fig. 6A). Mechanical strain significantly increased the basal phosphorylation of Akt and GSK-3β, which was partially blocked by the application of Akti-1/2 (Fig. 6B). Immunofluorescence staining results also showed that mechanical strain failed to induce nuclear accumulation of active β-catenin in the presence of Akti-1/2 (Fig. 6C). Therefore, mechanical strain-induced nuclear translocation of active β-catenin and osteogenesis need Akt activity and its downstream GSK-3β inhibition.

### Knockdown of PC1 on Strain-induced Activation of Intracellular Calcium and Akt/GSK-3β/β-catenin Axis

**Figure 7:**
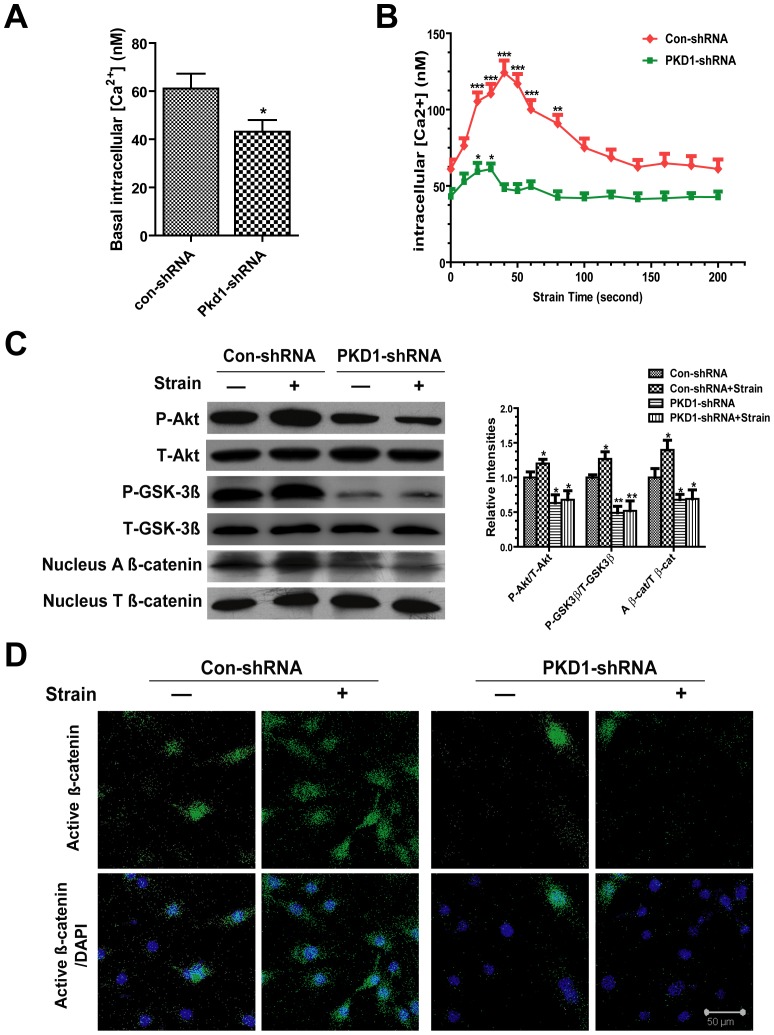
Knockdown of PC1 on strain-induced activation of intracellular calcium and Akt/GSK-3β/β-catenin axis. (A) Basal intracellular calcium levels in con-shRNA and PKD1-shRNA cells (n = 20). (B) Continuous measurement of intracellular calcium concentrations in con-shRNA (red) and PKD1-shRNA cells (green) after subjected to tensile strain. Strain-induced intracellular calcium responses significantly attenuated in Pkd1-shRNA cells compared with con-shRNA cells (n = 20). (C) Con-shRNA and PKD1-shRNA cells were subjected to 1 h tensile strain. Levels of the phospho-Akt, phospho-GSK-3β and nuclear active β-catenin were analyzed by western blots. The graph represented relative intensities of phospho-Akt, phospho-GSK-3β and nuclear active β-catenin bands normalized to their respective total protein, as measured by densitometry. (D) Con-shRNA and PKD1-shRNA cells were subjected to 1 h tensile strain before being fixed. Cellular distribution of active β-catenin (green staining) and nuclei counterstained with DAPI (blue staining) were determined by immunofluorescence and confocal microscopy. Scale bar, 50 μm. Each experiment was performed three times individually. Results are presented as the mean ± SEM, * indicates significantly different than controls (*, p<0.05; **, p<0.01; ***, p<0.001).

Compared with control shRNA cells, PKD1 shRNA cells displayed a lower basal intracellular calcium level (Fig. 7A). The intracellular calcium concentrations in con-shRNA and PKD1-shRNA cells were measured after subjected to mechanical strain once every 10 s. There was a significant increase in intracellular calcium concentrations in control shRNA cells, with a peak increased of about 2-fold roughly by 40 s relative to basal concentrations. Then the intracellular calcium level decreased slowly but maintained at moderate levels by 60–70 s before returning to baseline. In contrast, there was a significantly attenuated intracellular calcium curve in response to mechanical strain in PKD1 shRNA cells (Fig. 7B).

Basal phospho-Akt and phospho-GSK-3β levels were reduced in PKD1 shRNA cells compared with control shRNA cells, as well as the nuclear active β-catenin level. Deletion of PC1 blocked the strain-induced up-regulation of phospho-Akt, phospho-GSK-3β and nuclear active β-catenin after 1 h tensile strain in PKD1 shRNA cells (Fig. 7C). Furthermore, immunofluorescence staining analysis also showed that mechanical strain failed to promote nuclear accumulation of active β-catenin in PKD1 shRNA cells (Fig. 7D).

### Effects of Calcium Ionophore or LiCl on Osteoblastic Mechanoresponses in PC1 Deficient Osteoblasts under Strain Conditions

**Figure 8:**
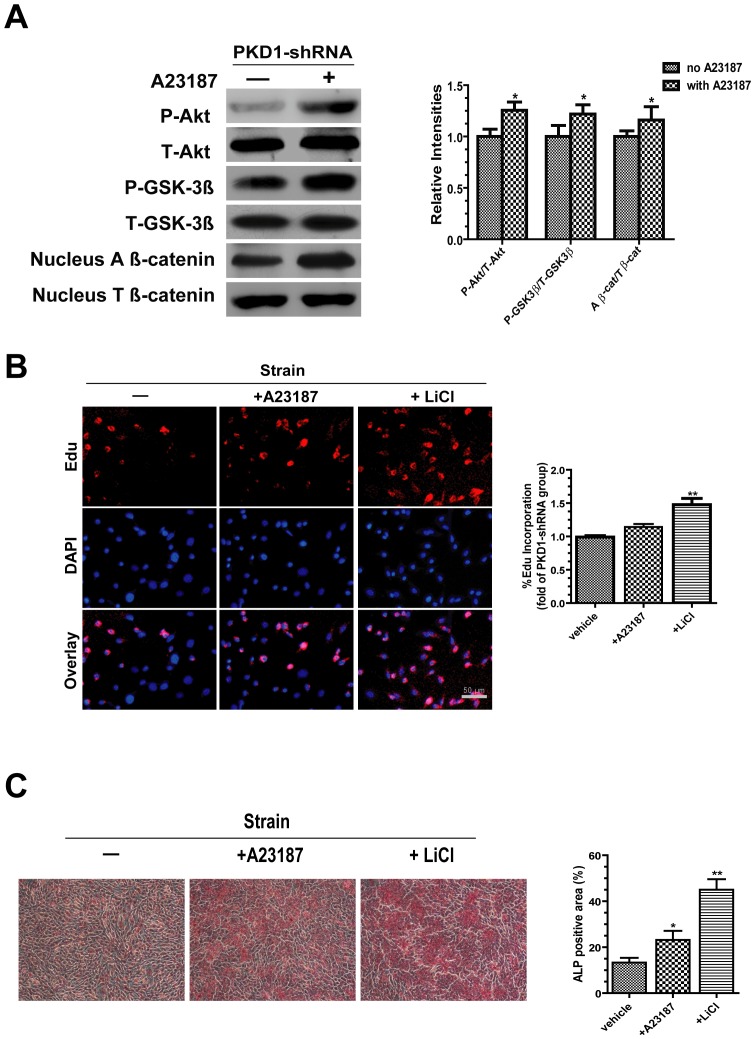
Effects of calcium ionophore or LiCl on osteoblastic mechanoresponses in PC1 deficient osteoblasts under mechanical strain conditions. (A) PKD1-shRNA cells were pre-incubated with A23187 (200 μM) for 1 h and then subjected to 1 h tensile strain. The phosphorylation of Akt, GSK-3β and the expression of nuclear active β-catenin were measured by western blots. (B) PKD1-shRNA cells were pre-incubated with A23187 (200 μM) or LiCl (20 mM) for 1 h and then subjected to 2 h tensile strain followed by EdU incorporation (20 μM) for 1 h. Analysis for proliferation rate of cells was carried out. (C) PKD1-shRNA cells were pre-incubated with A23187 (200 μM) or LiCl (20 mM) for 1 h. Cytochemical staining for alkaline phosphatase (ALP) was performed after 1 h tensile strain. Each experiment was performed three times individually. Results are presented as the mean ± SEM, * indicates significantly different than controls (*, p<0.05; **, p<0.01; ***, p<0.001).

Under mechanical strain conditions, applications of calcium ionophore A23187 in PKD1 shRNA cells [39], significantly up-regulated the phosphorylation of Akt, GSK-3β and the expression of nuclear active β-catenin compared with no A23187 control groups (Fig. 8A). Compared with control groups, applications of A23187 only slightly promoted PC1 deficient osteoblast proliferation, whereas treatment of LiCl significantly increased cell proliferation (Fig. 8B). Both applications of A23187 and LiCl caused a significant increase in ALP-positive colony areas compared with control groups (Fig. 8C). Our data manifest that intracellular calcium and Wnt/β-catenin pathway may play a role in PC1-mediated mechanoresponses in osteoblasts.

## Discussion

Bone remodels its mass and structure in response to mechanical loading. Mechanical stimuli in an optimal biological environment result in osteogenesis and proliferation [3]. Conversely, deficiency of physiological mechanical loading leads to osteopenia or osteoporosis. Therefore moderate physical exercise is critical for maintaining bone integrity and architecture [5]. However, the underlying physiological mechanisms of bone how to transform the environmental mechanical signals into biochemical signals remain poorly understood. Understanding the mechanisms should help identify new approaches for the treatment of musculoskeletal diseases and injuries. Here, in order to investigate the role of mechanosensory molecule PC1 in modulating strain-induced osteoblastic mechanoresponses, PKD1 gene was stably silenced in osteoblasts by using lentivirus-mediated shRNA technology. Our data demonstrated that continuous mechanical tensile strain significantly promoted osteoblastic differentiation and proliferation, but deficiency of PC1 impaired this promotion by suppressing intracellular calcium and downstream Akt/β-catenin/Runx2 signaling pathways for the first time.

PC1 is widely expressed in many kinds of tissues and cell types, certainly including bone [15], [40]. Recent studies show that loss of PC1 leads to seriously defective skeletogenesis [14], [41]. PC1 seems to be essential for the skeletal development through regulating Runx2, a key transcription factor in osteoblast differentiation [17], [18]. Here, PC1 deficient osteoblasts exhibited significantly impaired osteogenesis, which is consistent with the previous experimental results in vivo. Moreover, there is increasing evidence that PC1 acts as a mechanosensor that receives external signals and then transduces them into cellular responses in renal cells, such as cell proliferation, differentiation and morphology [13], [42], [43]. PKD1 gene expressed highly at various time points in osteoblasts after subjected to efficient mechanical strain, although changed scarcely. However, silence of PKD1 gene in osteoblasts exhibited an inhibited response to mechanical stimuli, resulting in an abolition of strain-induced up-regulation of osteogenic gene expressions and cell proliferation. Our findings suggest that PC1 plays an important role in skeletal responses to mechanical strain through regulating Runx2 in vitro, which is in accordance with recent work in vivo by Xiao et al [19].

Mechanical stimuli sensed by cells must be ultimately translated into biochemical changes in signaling events such as phosphorylation, transcription factor translocation or alterations of gene expression [9]. Our findings suggest that PC1 as a mechanosensory molecule is able to convert mechanical stimuli into biochemical responses that enhance osteogenesis via up-regulation of Runx2. However, the mechanisms involved in mechanical signaling between PC1 and strain-induced activity of Runx2 still remain unclear. Consequently, we focused on Wnt/β-catenin and Akt-dependent signaling pathways, because both contribute to bony responses to mechanical strain through direct regulation of Runx2, a target gene of β-catenin/TCF complex [38], [44], [45]. Furthermore, recent studies by Xiao et al. found that conditional PKD1 deletion mouse model displayed impaired differentiation and suppressed activation of PI3K-Akt-GSK3β-β-catenin signaling pathway in vivo [18], [46]. Indeed, our study further revealed that mechanical strain activated Akt, inhibited GSK3β, increased nuclear accumulation of active β-catenin and ultimately up-regulated Runx2. However, mechanical strain-induced activation of Wnt/β-catenin and Akt-dependent pathways disappeared in PC1 deficient osteoblasts, and the basal level of phospho-Akt, phospho-GSK3β, nuclear accumulation of active β-catenin and Runx2 also attenuated significantly. Applications of LiCl in PC1 deficient osteoblasts promoted cell differentiation and proliferation under mechanical strain conditions. Therefore, PC1 seems to function as a mechanosensor, which can sense mechanical stimuli, stimulate Akt-GSK-3β-β-catenin signaling pathway and then enhance Runx2 expression.

Interestingly, deletion of PC1 in MC3T3 cells restrained strain-induced proliferation, but significantly increased basal proliferation. In normal and adult kidney, PC1 functions by inducing the formation of PC1-tuberin-mTOR complex thereby inhibiting mTOR activity [47], yet in human ADPKD patients and mouse models, null of PC1 leads to the inability to assemble this inhibitory complex thereby causing mTOR pathway inappropriately activated [48], [49]. Further studies have provided strong evidences that PC1 controls the mTOR pathway in a Tsc2-dependent manner (inhibiting phosphorylation of tuberin) thus regulating cell growth and proliferation [50], [51]. Our data also supported this model indicating that this phenomenon is not tissue specific. Deletion of PC1 in osteoblasts may lead to inappropriate activation of mTOR pathway, finally resulting in increased cell proliferation, but these cells may have no osteogenic potential. Mechanical strain promoted osteoblats proliferation, but this strain-induced promotion disappeared in PC1 deficient osteoblasts. This phenomenon proves once again that PC1 function as a mechanosensory molecule to sense external mechanical stimuli. Once deletion of PC1, mechanically induced osteogenesis and proliferation were suppressed. Hou et al. provided evidences by using a mouse midpalatal suture expansion model, where PC1-deficient mice exhibited a significantly reduced osteogenic response to tensile stress across the suture, also demonstrating that the importance of PC1 in mechanical strain-induced osteoblastic differentiation and proliferation [52].

A rapid increase in intracellular calcium is the earliest response in mechanically stimulated bone cells [9], [27], which can be mobilized by several different forms of mechanical stimuli including membrane strain[53], pressure[32] and fluid flow [54]. PC1 located at primary cilium forms a mechanosensitive ion channel with polycystin-2 via its C-terminus and regulates calcium influx in response to fluid flow stimuli in renal cells [10], [12], [55]. Deletion of PC1 displayed much less calcium influx in response to fluid flow stimuli in human osteoblasts [56]. Our study also found that silence of PKD1 gene in MC3T3 cells caused a lower basal intracellular calcium level and an attenuated intracellular calcium curve in response to mechanical tensile strain stimuli. Furthermore, applications of calcium ionophore A23187 in PC1 deficient osteoblasts leaded to a significant increase in osteogenic differentiation and maturation, and partly reversed the blocking of mechanical strain-induced up-regulation of phospho-Akt, phospho-GSK-3β and nuclear active β-catenin expressions under mechanical strain conditions. Together, PC1-mediated intracellular calcium mobilization most likely subsequently stimulates downstream Akt-GSK-3β-β-catenin-Runx2 cascade and further links this stimulation to mechanical strain.

Recent work by Dalagiorgou et al. applied an antibody against the N-terminal of PC1 to uncover the role of PC1 as chief mechanosensory molecule in modulating osteoblastic gene expression [28]. Their data showed that stretching of human periodontal ligament fibroblasts pre-incubated with this antibody leaded to reduced expression of Runx2 through blocking Ca^2+^/NFAT signaling cascade. Human PDL cells isolated from the periodontal ligament might have stem cell-like features, possessing the multipotential to differentiate into osteoblast-like cells and chondrocytes [57], whereas they are still different from real osteoblasts. This study applied more efficient and controllable lentivirus-mediated shRNA technology to stably silence PKD1 gene in osteoblastic cell line. And our findings proved that PC1 mediates mechanical strain-induced not osteoblastic differentiation but proliferation via potentiation of intracellular calcium and downstream Akt/β-catenin signaling pathway. Dalagiorgou et al. and our researches both demonstrated that PC1 really functions as a mechanosensory molecule that mediates mechanical strain-induced osteoblastic mechanoresponses by activating intracellular calcium and via various downstream signaling pathways.

**Figure 9:**
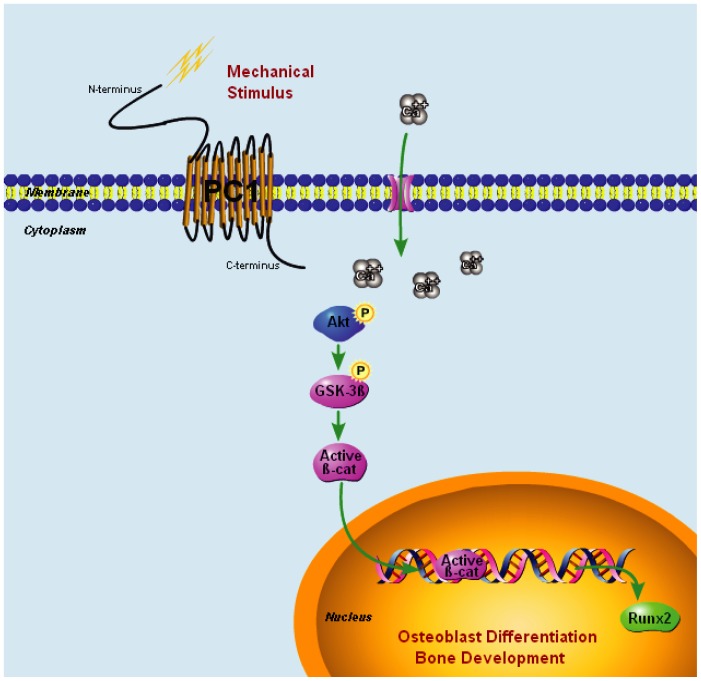
Model of PC1 mediating Akt-GSK-3β-β-catenin signaling pathway in osteoblast mechanotransduction. PC1 functions as a mechanosensory molecule that senses external mechanical stimuli, thereby converting these mechanical signals into potentiation of intracellular calcium. Then calcium-dependent Akt phosphorylates and then leads to the phosphorylation of GSK-3β. The phospho-GSK-3β inhibits β-catenin for proteolysis, which leads to active β-catenin nuclear translocation and transcriptional regulation of genes involved in osteoblastic proliferation and differentiation such as Runx2.

Collectively, the earliest events in osteoblast mechanotransduction are a rapid influx of extracellular Ca^2+^ and mobilization of intracellular Ca^2+^ [25], [57]. And mechanical strain also causes a rapid, transient accumulation of active β-catenin in the cytoplasm and its translocation to the nucleus, followed by up-regulation of Wnt/β-catenin target genes such as Runx2 [38], [58]. As depicted in Fig. 9, PC1 functions as a mechanosensor that senses external mechanical stimuli, thereby converting mechanical signals into potentiation of intracellular calcium. Then calcium-dependent Akt phosphorylates and leads to phosphorylation of GSK-3β. The phosphorylation of GSK-3β inhibits β-catenin for proteolysis, which leads to active β-catenin nuclear translocation and transcriptional regulation of genes involved in osteoblastic proliferation and differentiation such as Runx2 [44].

In conclusion, our findings present evidences that osteoblasts require mechanosensory molecule PC1 to sense mechainical strain inducing osteogenesis and proliferation, at least partially via potentiation of intracellular calcium and downstream Akt-GSK-3β-β-catenin signaling pathway. However, future work aims to determine whether PC1 participates in a mechanosensing complex with primary cilia and polycystin-2 and their potential mechanisms of mechanotransduction in osteoblast.
